# Evaluation of Serum microRNA Let-7c and Let-7d as Predictive Biomarkers for Metastatic Pancreatic Cancer

**DOI:** 10.5152/tjg.2022.21829

**Published:** 2022-08-01

**Authors:** Atike Gökçen Demiray, Aydın Demiray, Arzu Yaren, Burcu Yapar Taşköylü, Gamze Gököz Doğu, Serkan Değirmencioğlu, Umut Çakıroğlu, Nail Özhan, Canan Karan, Burçin Çakan Demirel, Tolga Doğan, Melek Özdemir

**Affiliations:** 1 Department of Medical Oncology, Pamukkale University Faculty of Medicine, Denizli, Turkey; 2 Department of Medical Genetics, Pamukkale University Faculty of Medicine, Denizli, Turkey

**Keywords:** MicroRNA let-7c, microRNA let-7d, pancreatic cancer

## Abstract

**Background:**

First-line treatments for metastatic pancreatic cancer are chemotherapy regimens consisting of 5-fluorouracil or gemcitabine; however, there are no biomarkers to help determine which patients might benefit from which treatment regimens. We aimed to show that microRNAs let-7c and 7d can be used as independent predictive biomarkers for metastatic pancreatic cancer.

**Methods:**

A total of 55 patients who had first-line chemotherapy with FOLFIRINOX or gemcitabine + capecitabine were included. Patients were divided into groups based on let-7c and let-7d levels and chemotherapy treatment as let-7c-7d high FOLFIRINOX, let-7c-7d high gemcitabine + capecitabine, let-7c-7d low FOLFIRINOX, and let-7c-7d low gemcitabine + capecitabine. Blood samples were taken from patients before chemotherapy for microRNA let-7c and 7d analysis. MicroRNA isolation was performed using a miRNeasy Serum/Plasma Kit and identified using spectrophotometric measurements. After isolation, microRNA was converted to cDNA using a microRNA cDNA Synthesis Kit with poly (A) polymerase tailing. The expression of microRNA was examined using quantitative real-time polymerase chain reaction.

**Results:**

The overall survival of patients who received FOLFIRINOX treatment with a high let-7c-7d level was statistically significantly longer than those who received gemcitabine + capecitabine with a high let-7c-7d level. In addition, patients with low let-7c expression receiving FOLFIRINOX progressed significantly 2.104 times earlier than patients with high let-7c expression receiving FOLFIRINOX.

**Conclusion:**

The serum MicroRNA let-7c level was found to be an independent predictive biomarker in the FOLFIRINOX treatment group.

**Main Points**

- Pancreatic cancer has an extremely poor prognosis due to late diagnosis, high metastatic potential, lack of effective treatment methods, and resistance to chemotherapy.
- Current treatments for metastatic pancreatic cancer are chemotherapy regimens with 5-fluorouracil or gemcitabine.
- There is no biomarker to predict which patients might benefit from which chemotherapy treatment regimens.
- MiRNA let-7c can be used as an independent predictive biomarker for FOLFIRINOX use in metastatic pancreatic cancer.

## Introduction

Pancreatic cancer (PC) is extremely aggressive and was the fourth leading cause of cancer deaths in the United States in 2020, with 57 600 new cases and 47 050 deaths reported.^1^ It is expected to be the second leading cause of cancer-related death in the United States by 2030.^2^ Due to late diagnosis, high metastatic potential, lack of effective treatment methods, and resistance to chemotherapy, PC has an extremely poor prognosis. At the time of diagnosis, 80% of patients are in the metastatic stage, for which the 5-year survival rate is 3%.^3^ Adenocarcinoma accounts for 90% of cases which are most commonly seen in the head region of the pancreas. Treatment for metastatic PC consists of chemotherapy regimens with FOLFIRINOX or gemcitabine (gemcitabine + erlotinib, gemcitabine + nab-paclitaxel, gemcitabine + capecitabine).^4^ Despite these treatments, the overall survival (OS) may be 1 year, and not all patients benefit from treatment.^5^ There are currently no biomarkers to predict which patients might benefit from which treatment regimen (except breast cancer gene 1-2 [BRCA1-2] and partner and localizer of BRCA2 [PALB2] mutations). To increase survival for metastatic cancer patients, the development of personalized treatment plans based on predictive markers provides the most appropriate and effective options.

MicroRNAs (miRNAs) are small, non-protein-coding ribonucleic acid molecules, approximately 19-24 nucleotides in length, which regulate gene expression at the translational level.^6^ A single miRNA can interact with multiple target genes with oncogenic or tumor suppressor functions and potentially regulate multiple cellular pathways.^7^ It is known that miRNA regulation of gene expression engages in many tumorigenic processes, including cell proliferation, migration, invasion, and metastasis.^8^ Studies have revealed the role of miRNAs in the diagnosis, proliferation, progression, metastasis, and chemotherapy resistance of various cancers.^9-12^ These study results have led to miRNAs becoming a focal point of molecular oncology. Differences in miRNA expression levels may be tumor-specific and in some cases, have been associated with prognosis.^13^ Highly expressed miRNAs can function as oncogenes by suppressing tumor suppressor genes, whereas poorly expressed miRNAs can function as tumor suppressor genes by negatively regulating oncogenes.^14^ These miRNAs are not only included in the cell content but can also be transported between cells via exosomes and can be found in serum. Let-7 is commonly known as a tumor suppressor, as it reduces cancer aggressiveness, chemotherapy resistance, and radioresistance. However, in rare cases, let-7 acts as an oncogene that increases cancer migration, invasion, chemotherapy resistance, and expression of genes associated with progression and metastasis.^15^ Let-7 abnormal regulation and expression have been associated with cancer initiation and progression, targeting many oncogenes. In PC, Kirsten ras sarcoma, signal transducer and activator of transcription 3, insulin-like growth factor-2 mRNA-binding protein, and high mobility group AT-hook 2(HMGA/HMGA2) are among the validated targets of let-7 .^16^ Because most of the current studies with miRNAs are in vitro, more studies are needed to determine their role in patients before they become predictive biomarkers and therapeutic targets in cancer treatment. Various miRNAs have been implicated in OS and disease-free survival and have been associated with tumor grade, metastasis, and tumor-node metastasis stage.^17,18^ Expression levels of various miRNAs such as miR-21, miR-196a-2, miR-155, and miR-210 are associated with poor PC prognosis.^19^ No clinical study is available with patient serum showing the relationship of let-7c and let-7d levels with OS and progression-free survival (PFS) in patients receiving FOLFIRINOX or gemcitabine + capecitabine (Gem + Cape) chemotherapy. Specific miRNAs that can be used as predictive markers for FOLFIRINOX are likely to exist. We aimed to determine whether miRNAs let-7c and 7d can be used as independent predictive biomarkers for metastatic PC.

## Materials and Methods

A total of 55 patients who were histopathologically diagnosed with pancreatic ductal adenocarcinoma and in the metastatic stage at the time of diagnosis were included in this study. As a control group, 38 healthy volunteers who did not have a chronic disease, any previous pathology of the pancreas (pancreatitis, cyst, benign neoplasia, operation, etc.), malignancy (pancreas or other), and did not use regular drugs were recruited. The median age of healthy individuals was 56 (43-65); 21 (55.26%) were male and 17 (44.73%) were female. The median age of the patients at diagnosis was 65.7 (34-87), 34 (61.8%) were male, 21 (38.18%) were female, 26 received FOLFIRINOX, and 29 received Gem + Cape treatment. The median age of patients receiving FOLFIRINOX treatment was 61 (34-82), while the median age of patients receiving Gem + Cape treatment was 69 (42-87). The performance score of the patients in both chemotherapy arms was 0-1. Written informed consent was obtained from patients in both groups. The study protocol was approved by the ethics committee of Pamukkale University Faculty of Medicine (Approval no: 60116787-020/27421, date: April 27, 2020).

### Study Design

The voluntary patients who were in de novo metastatic stage, with good performance scores (0-1), and who had first-line chemotherapy with FOLFIRINOX (5-fluorouracil 400 mg/m^2^ iv, 2400 mg/m^2^ iv infusion 1st day, calcium folinate 400 mg/m^2^ iv 1st day, oxaliplatin 85 mg/m^2^ iv 1st day, irinotecan 180 mg/m^2^ iv 1st day, every 14 days) or Gem + Cape (gemcitabine 1000 mg/m^2^ iv 1st, 8th, and 15th day, capecitabine 850 mg/m^2^ twice a day, every 14 days) were included in the study. Exclusion criteria were brain metastasis, performance score ≥ 2, secondary malignancy, previous chemotherapy, benign or malignant surgery of the pancreas, and adjuvant chemotherapy or radiotherapy due to pancreatic ductal adenocarcinoma. The patients’ age, gender, chronic diseases, drugs used, clinical tumor and lymph node stage, chemotherapy protocols, anatomical location of pancreatic ductal adenocarcinoma, and OS and time to progression were recorded from their clinical files. For our study, 7 mL of blood was taken from the patients and controls to study miRNAs before chemotherapy. Patients were compared after dividing into groups by low and high let-7c and let-7d level and FOLFIRINOX or Gem + Cape chemotherapy regime.

### Isolation of miRNA and Construction of cDNA

The sera obtained were treated with 1/5 of QIAzol, and miRNA isolation was performed using a miRNAs’ Serum/Plasma Kit (Qiagen cat: 217184 Hilden, Germany). The obtained miRNAs were evaluated by spectrophotometric measurements and stored at −20°C. After the isolation, miRNAs were converted to cDNA using an ABM miRNA cDNA Synthesis Kit with poly(A) polymerase tailing (ABM cat: G903 Richmond, Canada).

### Measurement of miRNA Expression Levels

Quantitative real-time polymerase chain reaction (qPCR) was performed using Eva-Green miRNA qPCR Master Mix (ABM, Richmond, Canada) as per the experimental protocol. The serum miRNA (hsa-let-7c and hsa-let-7d) expression level was determined for each patient and the control samples using miRNA qPCR Master Mix (ABM) kits. Target gene expressions were analyzed in the presence of cel-miR-39-3p expression levels in a CFX Connect Bio-Rad PCR machine (Bio-Rad, California, USA). All qPCR reactions were performed in duplicate to calculate the average values. The cycle threshold (Ct) was determined for each miRNA and a control (cel-miR-39-3p). The relative abundance of each miRNA transcript was then determined using the delta–delta Ct method. The delta–delta Ct was used to evaluate the relative expression levels of miRNA genes in the samples of patients and healthy controls.

### Statistical Analysis

Statistical analyses were performed using the Statistical Package for Social Sciences (SPSS) version 17 software (SPSS Inc.; Chicago, IL, USA). Descriptive data are expressed as medians and percentages. The Student’s *t*-test was used for continuous variables. Categorical variables were compared by a chi-square test (Fisher’s exact probability test). Survival curves were formed using the Kaplan–Meier compared by the log-rank test. The independent prognostic factors associated with patient survival (FOLFIRINOX and Gem + Cape) were evaluated using multivariate Cox proportional hazard regression analysis. Covariates included miR-7c and 7d serum levels, clinical nodal (N) and clinical tumor (T) status, and anatomic region of the pancreas (head, corpus, and tail). Statistical significance was defined as *P* < .05.

Using Ct values in the patient and healthy control groups, the increase or decrease ratio of expression levels of serum miRNA genes and the cel-miR-39-3p was calculated according to the following formula: 2^−el-m^ where △Ct = Ct target gene−Ct reference gene.

All data parameters were evaluated using the GraphPad Prism 7.0 program, applying unpaired *t*-tests and one-way analysis of variance. The results, clinicopathological factors, and prognoses were compared retrospectively between each miRNA high and low 2^−tes^ group. The same data sets were confirmed using the SPSS 17.0 software program.

## Results

The patients in the Gem + Cape group had more chronic diseases and drugs used, although not statistically significant, than those in the FOLFIRINOX group (*P* = .712). All patients in both treatment groups had at least 2 metastatıc lesions in liver, multiple intra-abdominal and mediastinal metastatic lymph nodes, and at least 1 bone metastasis. In addition, 2 patients had lung parenchymal metastases, 1 had adrenal metastases, and 1 had neck lymph node metastases. Patients receiving FOLFIRINOX received a median of 16 (12-30) cycles, while patients receiving Gem + Cape received a median of 14 (10-22) cycles.

**Figure 1.**
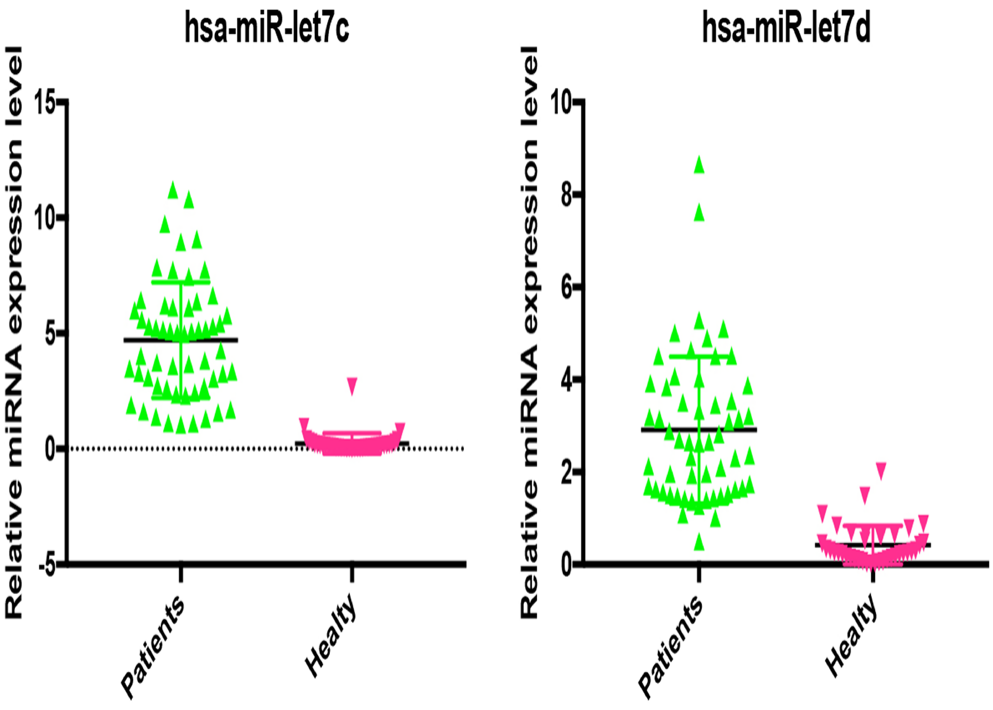
Serum miRNA let-7c and let-7d levels in healthy individuals and patients.

**Figure 2.**
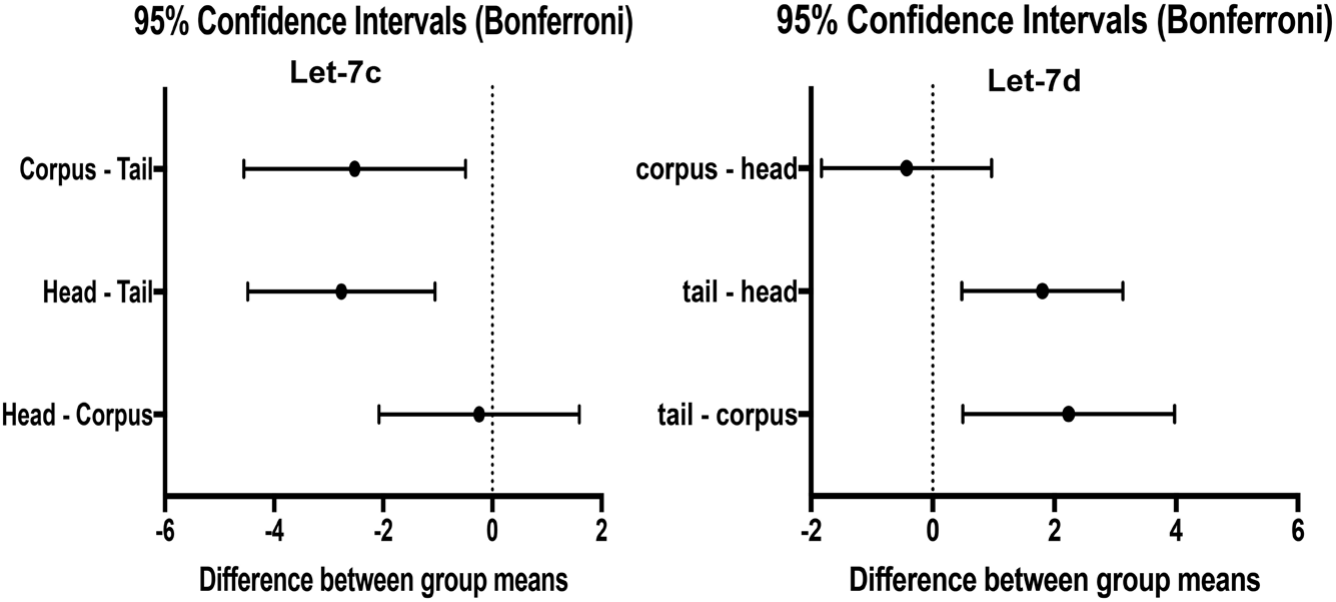
Serum miRNA let-7c and let-7d levels according to pancreatic cancer anatomic region of origin.

Serum miRNA let-7c and 7d levels of healthy individuals were statistically significantly lower than those of patients with PC (*P* < .05) (Figure 1). Let-7c and let-7d levels were statistically significantly higher in tail origin PC than head and body origins (*P* = .0006 let-7c head-tail, *P* = .001 let-7c body-tail, *P* = .0044 let-7d head-tail, *P* = .0077 let-7d body-tail) (Figure 2). There was no statistically significant difference between let-7c and let-7d levels between 65 years of age and under, gender, clinical tumor (cT), and lymph node (cN).

Of the patients, 11 were alive and 44 were deceased. The relationship between the numbers of let-7c and let-7d patients in the treatment groups and serum levels of let-7c and let-7d with median OS in the treatment groups is summarized in Table 1. The 1-year survival rate for all patients was 8.8%.

The relationship between the median OS and demographic and clinical findings in the let-7c and let-7d low and high patient groups is summarized in Table 2. The OS of the group with a high let-7c level < 65 years old was higher than the group with low let-7c, but it was not statistically significant. Although male patients with high let-7c had higher OS than patients with low let-7c, it was not statistically significant. Overall survival was higher in the group with let-7c higher than clinical T and N, although not statistically significant.

**Figure 3.**
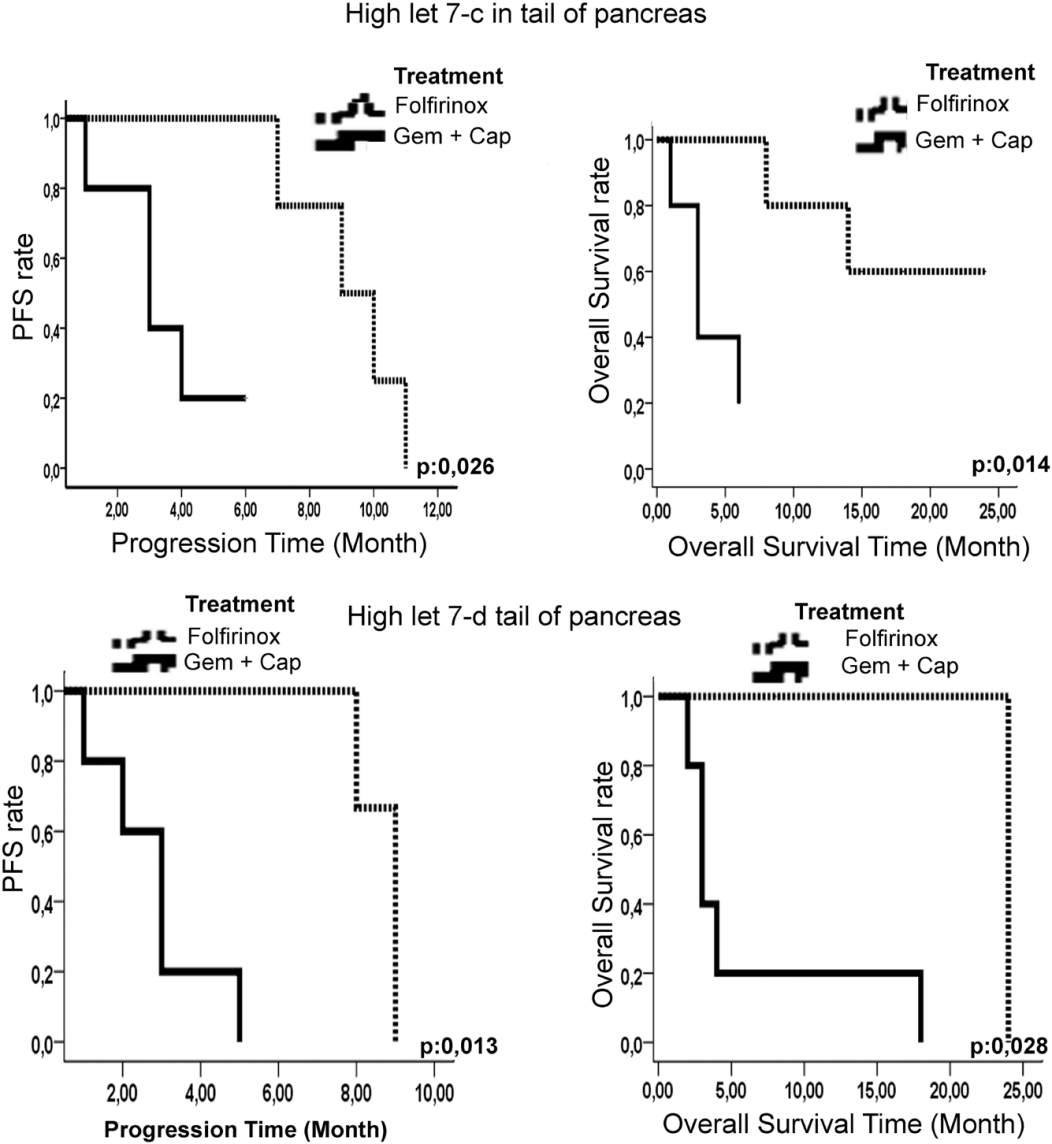
Progression-free survival (PFS) and overall survival (OS) of the high let-7c and let-7d Gem + Cape and FOLFIRINOX arms with pancreatic cancer of tail origin. Gem + Cape, gemcitabine + capecitabine.

The curves (Figure 3) and the relationship between the anatomical origin of PC and the median PFS and OS in the low and high let-7c and 7d groups receiving FOLFIRINOX and Gem + Cape are shown in Table 3 and Table 4, respectively. The median PFS of the let-7c and let-7d high Gem + Cape arm with a pancreatic tail origin is significantly shorter than the let-7c and let-7d high FOLFIRINOX arm. The median OS was also significantly less in the same group (*P* = .014 vs *P* = .028, respectively).

Based on the Cox proportional hazard model, multivariate survival analysis was performed using miRNA 7c and 7d serum levels (high vs low) and tail. The OS time was significantly dependent on miRNA 7c serum level (Table 4, *P* = .020) but not on 7d and tail. In the FOLFIRINOX group, the miRNA 7c serum level was an independent prognostic marker for PC patients, with a relative risk of 2.104 (Table 5).

## Discussion

In our study, serum miRNA let-7c and 7d levels were measured in patients with metastatic PC who had good performance scores and could receive chemotherapy. The association of these miRNAs with PFS and OS was evaluated in patients with low and high let-7c and let-7d levels receiving FOLFIRINOX or Gem + Cape treatment. We aimed to show that miRNA let-7c and let-7d might be used as independent predictive biomarkers in metastatic PC, which is extremely aggressive, resistant to chemotherapy, and has low survival. In the sense that this is the first clinical study conducted with miRNA let-7c and 7d in metastatic groups, evaluating serum levels and different treatment arms, our findings are important. In addition, we think our results will shed light on the findings of future studies. The power and effectiveness of the studies can be increased by conducting measurements in tumor tissue and cell lines simultaneously with serum levels.

A study of cholangiocarcinoma revealed the complex role of microRNA let-7c. Let-7c was expressed more in extrahepatic metastases and the serum of metastatic patients compared to those without metastases. In this study, overexpression of let-7c inhibited cancer’s invasive capacity in vitro while increasing distant metastasis capacity in vivo. Let-7c has been shown to directly target the enhancer of zeste 2 polycomb repressive complex 2 subunit (EZH2) and disheveled segment polarity protein 3 (DVL3) genes. This dual role in the regulation of cholangiocarcinoma could be mimicked by the regulation of EZH2 and DVL3 expression. Therefore, their research proposes miRNA 7c as a new biomarker for identifying patients with metastatic disease, providing strong experimental evidence for the involvement of let-7c in the capacity for distant metastasis of cholangiocarcinoma.^20^ Our patients were in the metastatic stage and had high let-7c levels when compared to the healthy group. In many studies, let-7 family expression was low in PC cell lines, operated or locally advanced stages.^21^ Still, there is no study in the literature with the let-7 family from metastatic stage PC lines, serum, or metastatic tissues. Therefore, it should be determined whether let-7 has a relationship with EZH2 and DVL3 genes and whether it has a dual role in the metastatic process in PC.

Researchers suggest that miRNAs play a crucial role in regulating chemotherapy sensitivity in PC.^22^ In a preclinical study in PC cell lines, both miR-211 and let-7 increased sensitivity to gemcitabine by decreasing the expression of its target ribonucleotide reductase regulatory subunit M2(RRM2), an important target of gemcitabine, and also by inhibiting RRM2 or activating let-7.^23^ It has been shown that PC cells can reverse the chemoresistance to gemcitabine.^24^ In our clinical study, there was no statistically significant difference in survival in the low and high let-7c and 7d Gem + Cape groups. Thus, it was hypothesized that low or high let-7c and 7d might not be associated with gemcitabine sensitivity or resistance.

Why patients with high let-7c and 7d levels respond better to FOLFIRINOX than to Gem + Cape is one of the issues that needs to be investigated. Among the possible protein targets of let-7c and 7d in the in-silicone database is the high mobility group at-hook 2 (HMGA2) protein. In the literature, HMGA2 protein is one of the let-7c and 7d expression targets in studies conducted on ovarian, colon, breast, and lung cancer cell lines. The increase in let-7 expression causes the expression of HMGA2 protein to be suppressed.^25,26^ Another study conducted in colorectal cancer cell lines showed that decreased HMGA2 protein expression causes sensitivity to 5-FU.^27^ In our study, the OS and PFS were significantly higher in FOLFIRINOX patients with high let-7c and 7d levels, suggesting that HMGA2 protein was suppressed. As a result, sensitivity to FOLFIRINOX might be increased. Therefore, we predict that let-7c and 7d may be predictive biomarkers for 5-fluorouracil-based therapies. In addition, the increase of let-7c and 7d expression in PC of tail origin is an issue that needs to be examined histopathologically. Differences in results according to PC anatomical origin may occur because they contain heterogeneous cell populations such as ductal, acinar, and islet cells, together with inflammatory and fibroblastic cells that play a role in tumor development.^28^

There were some limitations in our study. FOLFIRINOX, gemcitabine + nab-paclitaxel, and gemcitabine + erlotinib are strongly recommended as first-line treatment in patients with metastatic pancreatic adenocarcinoma with good performance, so we preferred FOLFIRINOX treatment in patients with good performance. The Gem + Cape combination is also recommended for patients with good performance, although the level of evidence is different from FOLFIRINOX. We also preferred Gem + Cape as a first-line treatment for patients with good performance, higher average age compared to the other group, more chronic diseases and drugs used, being accessible and indicated in our country, and possible toxicities that may cause more morbidity and mortality. Since most of the patients received chemotherapy treatments in other centers and applied to us for response evaluation, side-effect assessment could not be performed adequately in most patients. For this reason, data about side effects could not be shared. As it is known, most patients with metastatic PC cannot tolerate chemotherapy because their performance scores are poor. These patients are usually given palliative support therapy. Since this group comprised the majority of our patients, only a small number of patients could be included in the study.

## Conclusion

Our results suggest that high let-7c and 7d levels might be related to the metastatic process. High levels of let-7c and 7d may indicate PC’s aggressiveness and a good response to FOLFIRINOX therapy. We propose that let-7c can be used as a predictive marker for FOLFIRINOX treatment. Clinical studies with larger populations and explanations of molecular mechanisms are needed to support this hypothesis.

## Figures and Tables

**Table 1.** The Relationship of Pancreatic Cancer Median Overall Survival for High- and Low-Level Serum let-7c and 7d with FOLFIRINOX and Gemcitabine + Capecitabine Treatment Groups

Median Overall Survival (Months)									
Chemotherapy Regimens	n	hsa-let-7c			hsa-let-7d			Total	*P*
Low (26)	High (29)	*P*	Low (32)	High (23)	*P*
FOLFIRINOX	26	9.1 ± 1.7 (12)	13.37 ± 2.3 (14)*	.343	11.1 ± 1.6 (15)	13.1 ± 3.1 (11)**	.845	11.8 ± 1.7	**.001**
Gemcitabine + vapecitabine	29	4.2 ± 1.1 (14)	5.7±1.2 (15)*	.372	6.1 ± 1.4 (17)	6.53 ± 1.95 (12)**	.694	5.04 ± 0.8
Total	55	6.30 ± 1	10.6 ± 1.6	**.039**	7.37 ± 0.9	11.62 ± 2.2	**.049**		

*P = .018, ** *P =* 0.052.

**Table 2.** The Relationship Between Median Overall Survival and Demographic and Clinical Findings in Serum High and Low Let-7c and Let-7d Level Groups

Median Overall Survival (months)							
Factor	n	hsa-let-7c			hsa-let-7d		
(55)	Low (26)	High (29)	*P*	Low (32)	High (23)	*P*
Age							
≥65	32	6.53 ± 1.31 (15)	6.31 ± 1.2 (17)	.935	6.73 ± 1.23 (19)	6 ± 1.5 (13)	.291
<65	23	6.36 ± 1.8 (11)	13.77 ± 2.75 (12)	.080	9.23 ± 1.97 (13)	9.78 ± 2.7 (10)	.197
Sex							
Male	21	4.8 ± 1.10 (10)	10.73 ± 2.23 (11)	.185	7.5 ± 1.39 (12)	7.4 ± 2.38 (9)	.913
Female	34	7.5 ± 1.53 (16)	7.73 ± 1.56 (18)	.556	7.56 ± 1.55 (20)	7.14 ± 1.64 (14)	.770
cT *							
cT1/T2/T3	34	6.78 ± 1.43 (14)	8.59 ± 1.84 (20)	.945	6.68 ± 1.14 (21)	10.90 ± 2.78 (13)	.185
cT4	21	8.59 ± 1.84 (12)	10.11 ± 2.08 (9)	.391	9.45 ± 2.14 (11)	4.8 ± 0.77 (10)	.106
cN**							
cN1	27	6.06 ± 1.34 (15)	7.64 ± 1.55 (12)	.621	7.4 ± 1.39 (14)	5.63 ± 1.96 (14)	.213
cN2	28	7.0 ± 1.76 (11)	9.85 ± 2.03 (17)	.884	7.63 ± 1.6 (18)	9.21 ± 2.15 (9)	.918

*Clinical tumor status, **Clinical lymph node status.

**Table 3.** The Relationship Between Median Progression-Free Survival and the Anatomical Origin of Pancreatic Cancer in Serum Low and High-Level Let-7c and 7d FOLFIRINOX and Gemcitabine + Capecitabine Treatment Groups

Factor	Median Progression-Free Survival (Months)					
	High			Low		
FOLFIRINOX	Gems + Cape*	P**	FOLFIRINOX	Gem + Cape*	P**
Hsa-let-7c						
Head	5.75 ± 1.2	4.87 ± 1.3	.640	6.286 ± 1.9	3.1 ± 0.7	.168
Body	11 ± 2.8	4 ± 0	.083	7.5 ± 5.5	7.2 ± 0	.650
Tail	9.25 ± 0.8	3.4 ± 0.7	**.026**	10.5 ± 1.5	5.2 ± 1.5	.153
Hsa-let-7d						
Head	5 ± 1.43	5 ± 2.01	.620	7 ± 1.7	3.63 ± 0.9	.136
Body	5.5 ± 1.5	3.66 ± 1.4	.321	9.25 ± 2.5	3.5 ± 0.5	.207
Tail	8.66 ± 0.3	2.8 ± 0.66	**.013**	11.5 ± 0.5	4 ± 0.9	.113

*Gemcitabine + capecitabine, **P < .05 is statistically significant.

Gem + Cape, gemcitabine + capecitabine.

**Table 4.** The Relationship Between Median Overall Survival and Anatomical Origin of Pancreatic Cancer in Low- and High-Level Serum Let-7c and 7d with FOLFIRINOX and Gemcitabine + Capecitabine Treatment

Factor	Median Overall Survival (Months)					
	High			Low		
FOLFIRINOX	Gem + Cape*	P**	FOLFIRINOX	Gem + Cape*	P**
Hsa-let-7c						
Head	6.92 ± 1.25	5.75 ± 1.8	.530	7.85 ± 1.8	3.9 ± 1.4	.077
Body	12 ± 0	7 ± 3	.225	7.5 ± 5.5	7.2 ± 0	.650
Tail	18.8 ± 2.9	3.8 ± 1	**.014**	10 ± 2.44	5.2 ± 1.6	.096
Hsa-let-7d						
Head	6.54 ± 1	8.3 ± 4.78	.923	10.12 ± 2.3	5.43 ± 1.8	.185
Body	5.5 ± 1.5	5 ± 1	.513	11 ± 2.6	3.5 ± 0	.207
Tail	24 ± 0	6 ± 3.01	**.028**	14 ± 0	5.6 ± 1.6	.212

*Gemcitabine + capecitabine, **P < .05 is statistically significant.

Gem + Cape, gemcitabine + capecitabine.

**Table 5.** Multivariate Survival Analysis (Cox Regression Model) of Clinical Prognostic Factors and Let-7c and 7d Levels in Patients with FOLFIRINOX

Factor	Relative Risk	95% CI	P*
Let-7c	2.104	1.125-3.933	**.020**
Let-7d	1.876	0.987-3.567	.055
Tail (7c)	1.826	0.643-5.186	.240
Tail (7d)	2.382	0.755-7.508	.155

*P < .05 is statistically significant.
